# Detection of patent ductus arteriosus with intraoperative transesophageal echocardiography in a patient undergoing closure of coronary artery to pulmonary artery fistula

**DOI:** 10.1186/s40981-017-0132-3

**Published:** 2017-12-08

**Authors:** Yuka Miyata, Yukio Hayashi

**Affiliations:** Anesthesiology Service, Sakurabashi-Watanabe Hospital, 2-4-32, Kita-ku, Osaka, 530-0001 Japan

**Keywords:** Coronary artery fistulas, Patent ductus arteriosus, Shunt ratio, Transesophageal echocardiography

## Abstract

**Background:**

Coronary artery to pulmonary artery fistula is an unusual vascular anomaly, and the shunt ratio of this fistula is usually small.

**Case presentation:**

We report anesthetic management of a 55-year-old female with annuloaortic ectasia, aortic valve regurgitation, and coronary artery to pulmonary artery fistula undergoing radical repair. We calculated the left-to-right shunt ratio after placement of a pulmonary artery catheter and found that the ratio was unexpectedly high. Thus, we explored the presence of another shunt by intraoperative transesophageal echocardiography and found patent ductus arteriosus undiagnosed before operation.

**Conclusions:**

A combination of a pulmonary artery catheter and transesophageal echocardiography is useful to explore the presence of another shunt, such as patent ductus arteriosus during anesthesia.

## Background

Coronary artery to pulmonary artery fistula is an unusual vascular anomaly, and the shunt ratio of this fistula is usually small [1, 2]. This anomaly is also known to be associated with various congenital heart diseases, such as tetralogy of Fallot and patent ductus arteriosus (PDA) [3, 4]. In this case, we calculated the left-to-right shunt ratio after placement of a pulmonary artery catheter in a patient undergoing closure of coronary artery to pulmonary artery fistula, and the ratio was unexpectedly large. Thus, we explored another shunt with intraoperative transesophageal echocardiography (TEE) and found PDA undiagnosed before operation.

## Case presentation

Written patient consent was obtained and our institutional ethical committee approved publication of this case report.

A 55-year-old female (157 cm tall and weighing 58 kg) was admitted to our hospital for annuloaortic ectasia (AAE) and moderate aortic valve regurgitation (AR). The preoperative transthoracic echocardiography (TTE) examination confirmed AAE and moderate AR and detected abnormal blood flow just superior to the pulmonary valve. A coronary angiography demonstrated two fistulas from the right coronary cusp and left coronary artery with contrast draining to the pulmonary artery. Thus, the patient was diagnosed with AAE, AR, and coronary artery to pulmonary artery fistula and scheduled for repairing the dilated aortic root while preserving the native aortic valve and closure of the fistula.

**Fig. 1:**
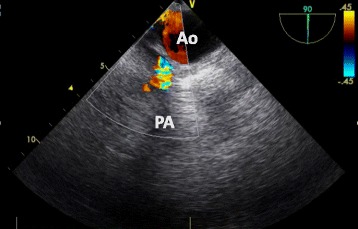
Perioperative transesophageal echocardiography. Abnormal blood flow contrast draining from the descending aorta to the pulmonary trunk with upper esophageal aortic arch short-axis view

Anesthesia was induced with midazolam, 10 mg; fentanyl, 0.2 mg; and vecuronium, 8 mg and maintained with propofol, remifentanil, and vecuronium. After induction of anesthesia, a pulmonary artery catheter was placed through the right internal jugular vein and we performed blood sampling from the pulmonary artery, the right atrium, and the radial artery. We detected the oxygen step-up in the pulmonary artery and calculated that pulmonary flow/systemic flow (Qp/Qs) and left-to-right shunt ratio was 1.9 and 46%, respectively (oxygen saturation in the radial artery, the right atrium, and the pulmonary artery is 99.6, 73.3, and 85.5, respectively). Furthermore, we confirmed shunt flow of the coronary artery to pulmonary artery fistula draining to just superior to the pulmonary valve by TEE. Since the calculated values of Qp/Qs and left-to-right shunt ratio were unexpectedly high, we explored the presence of another shunt which had not been detected by preoperative TTE or plain computed tomography. Then, we found abnormal blood flow contrast draining from the descending aorta to the pulmonary trunk with upper esophageal aortic arch short-axis view (Fig. 1). We suspected the presence of PDA and reported it to surgeons, and then, closure of PDA was added to the schedule.

After establishment of cardiopulmonary bypass (CPB), the fistula from the right coronary cusp was found easily on the heart and it drained to the pulmonary artery. We tried to snare the fistula and confirmed no ECG changes, and then, we ligated this fistula. Then, the aorta was cross-clamped and the heart was arrested. The surgical findings after incision of the pulmonary main truncus included two orifices at just superior to the pulmonary valve and at the roof of the pulmonary trunk. The former orifice was drained from the fistula from the right and left coronary artery. After cardiac arrest, significant blood flow was noted through the latter orifice, so we considered that the latter orifice was drained from PDA. These orifices were closed, and then, repairing the dilated aortic root while preserving the native aortic valve was performed. Weaning from CPB was uneventful. We confirmed no residual shunt by TEE and calculated Qp/Qs as 1.0.

## Discussion

Coronary artery to pulmonary artery fistula is a relatively unusual congenital anomaly and occurs in about 0.2 to 0.4% of patients with a congenital heart disease [1, 3, 4]. It has been reported that this anomaly is found in 0.3~1.3% of patients undergoing coronary angiography [2, 3, 5]. It has been reported that patients with this anomaly is also found in association with another congenital anomaly, including atrial septal defect, tetralogy of Fallot, PDA, ventricular septal defect, pulmonary atresia, and so on [1, 4].

When the drainage site of the coronary fistula is the pulmonary artery, the shunt ratio is generally small [2]. However, in the present case, the shunt ratio after induction of anesthesia was 46%, suggesting the presence of another shunt which had not been detected before operation; so, we explored shunt blood flow around the pulmonary artery and cardiac chambers and find PDA. Although the application of the pulmonary artery catheter during cardiac surgery is controversial [6], the catheter was useful in this case, because calculation of Qp/Qs and the shunt ratio was done easily and it may be a good trigger to explore another shunt undiagnosed before operation.

The echocardiographic diagnosis of PDA was made by direct visualization of a shunt flow in the duct draining from the descending aorta to pulmonary trunk. However, it is usually difficult to show clearly the structure of PDA and the flow by TTE in adult patients, because PDA is far from the transducer on the chest wall and there is interference from lung tissue and ribs. On the contrary, TEE has a much better window to the posterior structures of thorax. A previous report by Shyu et al. [7] demonstrated that TEE was more sensitive and specific in the diagnosis of PDA than TTE in adult patients. Thus, we should not hesitate to explore the presence of PDA, even if we did not diagnose PDA with preoperative TTE.

## Conclusions

The placement of a pulmonary artery catheter is useful to calculate Qp/Qs and the shunt ratio in patients with left-to-right shunt after induction of anesthesia, and we should explore the presence of another shunt, such as PDA, with TEE in patients with coronary fistulas, when Qp/Qs and shunt ratio after induction of anesthesia is unexpectedly high.

## Abbreviations

AAE: Annuloaortic ectasia
AR: Aortic valve regurgitation
CPB: Cardiopulmonary bypass
PDA: Patent ductus arteriosus
Qp/Qs: Pulmonary flow/systemic flow
TEE: Transesophageal echocardiography
TTE: Transthoracic echocardiography
